# Methyl 2-(7-benz­yloxy-1-naphth­yl)-2-oxoacetate

**DOI:** 10.1107/S160053680801982X

**Published:** 2008-07-05

**Authors:** Hoong-Kun Fun, Suchada Chantrapromma, Shu-Xian Li, Hua-Min Li

**Affiliations:** a X-ray Crystallography Unit, School of Physics, Universiti Sains Malaysia, 11800 USM, Penang, Malaysia; b Crystal Materials Research Unit, Department of Chemistry, Faculty of Science, Prince of Songkla University, Hat-Yai, Songkhla 90112, Thailand; c Department of Chemistry, Beijing Normal University, Beijing 100875, People’s Republic of China

## Abstract

In the crystal structure of the title compound, C_20_H_16_O_4_, the naphthalene ring system makes dihedral angles of 43.79 (7) and 83.70 (9)°, respectively, with the mean planes of the phenyl ring and the acetate unit. C—H⋯π inter­actions involving all the aromatic six-membered rings are observed. The mol­ecules are stacked into columns along the *a* axis and adjacent columns are linked by weak C—H⋯O inter­actions.

## Related literature

For related literature on hydrogen-bond motifs, see: Bernstein *et al.* (1995 ▶). For values of bond lengths, see: Allen *et al.* (1987 ▶). For related literature on bioactivities of compounds containing aromatic rings, see, for example: Hartwig (1998 ▶); Knepper *et al.* (2004 ▶); Kunz *et al.* (2003 ▶); Ley & Thomas (2003 ▶); Palucki *et al.* (1997 ▶).
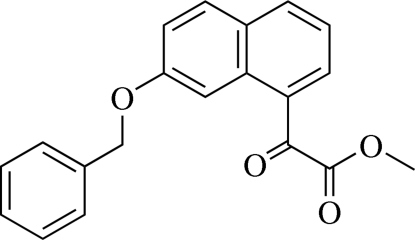


## Experimental

### 

#### Crystal data

C_20_H_16_O_4_
*M* *_r_* = 320.33
Orthorhombic, 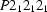

*a* = 5.6145 (3) Å
*b* = 15.7422 (8) Å
*c* = 17.3843 (8) Å
*V* = 1536.50 (13) Å^3^
*Z* = 4
Mo *K*α radiation
μ = 0.10 mm^−1^
*T* = 100.0 (1) K
0.58 × 0.32 × 0.10 mm

#### Data collection

Bruker SMART APEXII CCD area-detector diffractometer
Absorption correction: multi-scan (*SADABS*; Bruker, 2005 ▶) *T*_min_ = 0.946, *T*_max_ = 0.991
17379 measured reflections
2575 independent reflections
2380 reflections with *I* > 2σ(*I*)
*R*_int_ = 0.038

#### Refinement

*R*[*F* ^2^ > 2σ(*F* ^2^)] = 0.034
*wR*(*F* ^2^) = 0.100
*S* = 1.08
2575 reflections
218 parameters
H-atom parameters constrained
Δρ_max_ = 0.29 e Å^−3^
Δρ_min_ = −0.17 e Å^−3^

### 

Data collection: *APEX2* (Bruker, 2005 ▶); cell refinement: *APEX2*; data reduction: *SAINT* (Bruker, 2005 ▶); program(s) used to solve structure: *SHELXTL* (Sheldrick, 2008 ▶); program(s) used to refine structure: *SHELXTL*; molecular graphics: *SHELXTL*; software used to prepare material for publication: *SHELXTL* and *PLATON* (Spek, 2003 ▶).

## Supplementary Material

Crystal structure: contains datablocks global, I. DOI: 10.1107/S160053680801982X/is2310sup1.cif

Structure factors: contains datablocks I. DOI: 10.1107/S160053680801982X/is2310Isup2.hkl

Additional supplementary materials:  crystallographic information; 3D view; checkCIF report

## Figures and Tables

**Table 1:** Hydrogen-bond geometry (Å, °) *Cg*1, *Cg*2 and *Cg*3 are the centroids of the C1–C4/C9–C10, C4–C9 and C12–C17 rings, respectively.

*D*—H⋯*A*	*D*—H	H⋯*A*	*D*⋯*A*	*D*—H⋯*A*
C10—H10*A*⋯O2	0.93	2.28	2.896 (2)	124
C14—H14*A*⋯O2^i^	0.93	2.52	3.315 (2)	144
C20—H20*B*⋯O1^ii^	0.96	2.53	3.458 (2)	163
C7—H7*A*⋯*Cg*2^iii^	0.93	3.15	3.8529 (18)	134
C13—H13*A*⋯*Cg*3^iv^	0.93	3.13	3.8070 (19)	132
C17—H17*A*⋯*Cg*1^v^	0.93	3.12	4.0033 (17)	159

Symmetry codes: (i) 
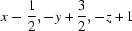
; (ii) 
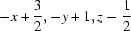
; (iii) 
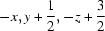
; (iv) 
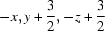
; (v) 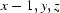.

**Fig. 1.**
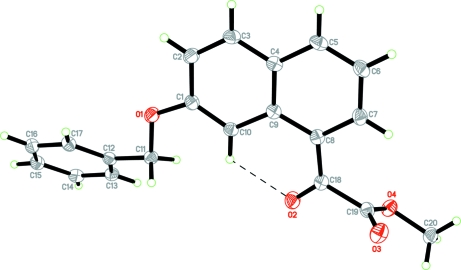
The asymmetric unit of (I), showing 50% probability displacement ellipsoids and the atomic numbering. The weak C—H···O intramolecular interaction is shown as a dashed line.

**Fig. 2.**
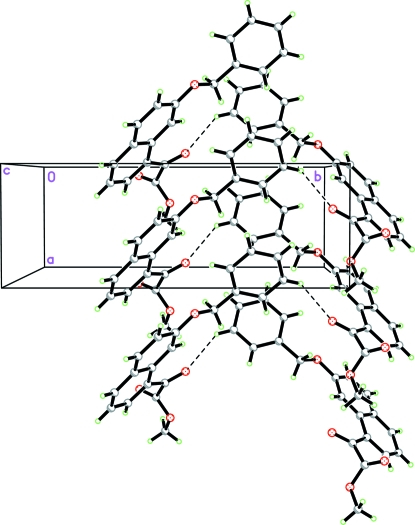
The crystal packing of (I), viewed down the c axis. Hydrogen bonds are shown as dashed lines.

## supplementary crystallographic information

### Comment

Ether compounds containing aromatic rings are useful intermediates in organic synthesis and are found in a large number of biologically active compounds. Some ethers containing aromatic units such as perrottetines, riccardin B and marchantin quinone exert considerable pharmacological activities, such as influencing blood coagulation. Others found usage as antifungal peperazinomycin and the glycopeptide antibiotics vancomycin (Hartwig, 1998; Knepper *et al.*, 2004; Kunz *et al.*, 2003; Ley & Thomas, 2003; Palucki *et al.*, 1997). Williamson reaction is a useful method to prepare ether compounds. In the case of the reaction between phenol and alkyl halide, phenols readily react with a mild base like potasuim carbonate to form phenoxide ions, which then substitute the –*X* group in the alkyl halide, forming an ether with an aryl group attached to it. In our ongoing project to synthesize novel ether compounds which can be used for biological research, we report herein the synthesis and crystal structure of the title compound, (I).

In the asymmetric unit of (I) in Fig. 1, the naphthalene ring is planar, with a maximum deviation of 0.0184 (18) Å for atom C1. The dihedral angle between the phenyl and naphthalene rings is 43.79 (7)°. The benzyloxy group (O1/C11–C17) is (-)anti-periplanar (-*ap*) and attached to the C1–C4/C9–C10 ring with C1—O1—C11—C12 torsion angle of -170.20 (14)°. Atoms O3, O4, C19 and C20 lie on the one plane whereas atoms O2, C8, C18 and C19 lie on the another plane. The dihedral angle between these two planes is 73.14 (12)°. The dihedral angle between the mean plane through the O3/O4/C19/C20 plane and naphthalene ring is 83.70 (9)°. The conformation of the oxyacetate unit is (-)anti-clinal (-*ac*) with C8—C18—C19—O4 torsion angle of -108.69 (16)°. A weak C10—H10A···O2 interaction generates a S(6) ring motif (Bernstein *et al.*, 1995). Bond distances and angles have normal values (Allen *et al.*, 1987).

The crystal packing of (I) in Fig. 2, shows that the molecules are stacked into column along the *a* axis and the adjacent columns were linked by weak C—H···O interactions. The crystal is stabilized by C—H···π interactions (Table 1); *Cg*_1_, *Cg*_2_ and *Cg*_3_ are the centroids of C1–C4/C9–C10, C4–C9 and C12–C17 rings, respectively.

### Experimental

The title compound was synthesized by stirring a mixture of methyl 2-(2-hydroxynaphthalen-8-yl)-2-oxoacetate (1.0 g, 4.3 mmol), 3-A° molecular sieves (2.0 g), potassium carbonate (0.7 g, 5.1 mmol) and benzyl bromide (0.75 g, 4.4 mmol) in dry DMF (35 ml) for 24 h, after which it was filtered and the filtrate evaporated. The residue was recrystallized from ether–n-hexane mixture (2:1 *v*/*v*) to give the desired compound (I) (1.10 g, 80% yield). Single crystals suitable for X-ray diffraction analysis were obtained by slow evaporation of the solvent from an ether–n-hexane solution (m.p. 358 K).

### Refinement

All H atoms were placed in calculated positions, with C—H = 0.93 Å and *U*_iso_(H) = 1.2*U*_eq_(C) for aromatic, C—H = 0.97 Å and *U*_iso_(H) = 1.2*U*_eq_(C) for CH_2_, and C—H = 0.96 Å and *U*_iso_(H) = 1.5*U*_eq_(C) for CH_3_ atoms. A rotating group model was used for the methyl groups. A total of 1721 Friedel pairs were merged before final refinement as there is no large anomalous dispersion for the determination of the absolute configuration.

### Crystal data

**Table:**

C_20_H_16_O_4_	*D*_x_ = 1.385 Mg m^−^^3^
*M**_r_* = 320.33	Melting point: 358 K
Orthorhombic, *P*2_1_2_1_2_1_	Mo *K*α radiation λ = 0.71073 Å
Hall symbol: P 2ac 2ab	Cell parameters from 2575 reflections
*a* = 5.6145 (3) Å	θ = 1.8–30.0º
*b* = 15.7422 (8) Å	µ = 0.10 mm^−^^1^
*c* = 17.3843 (8) Å	*T* = 100.0 (1) K
*V* = 1536.50 (13) Å^3^	Plate, colourless
*Z* = 4	0.58 × 0.32 × 0.10 mm
*F*_000_ = 672	

### Data collection

**Table:**

Bruker SMART APEXII CCD area-detector diffractometer	2575 independent reflections
Radiation source: fine-focus sealed tube	2380 reflections with *I* > 2σ(*I*)
Monochromator: graphite	*R*_int_ = 0.039
Detector resolution: 8.33 pixels mm^-1^	θ_max_ = 30.0º
*T* = 100.0(1) K	θ_min_ = 1.8º
ω scans	*h* = −7→7
Absorption correction: multi-scan(SADABS; Bruker, 2005)	*k* = −22→22
*T*_min_ = 0.946, *T*_max_ = 0.991	*l* = −22→24
17379 measured reflections	

### Refinement

**Table:**

Refinement on *F*^2^	Secondary atom site location: difference Fourier map
Least-squares matrix: full	Hydrogen site location: inferred from neighbouring sites
*R*[*F*^2^ > 2σ(*F*^2^)] = 0.034	H-atom parameters constrained
*wR*(*F*^2^) = 0.100	*w* = 1/[σ^2^(*F*_o_^2^) + (0.0565*P*)^2^ + 0.2831*P*] where *P* = (*F*_o_^2^ + 2*F*_c_^2^)/3
*S* = 1.08	(Δ/σ)_max_ = 0.001
2575 reflections	Δρ_max_ = 0.29 e Å^−^^3^
218 parameters	Δρ_min_ = −0.17 e Å^−^^3^
Primary atom site location: structure-invariant direct methods	Extinction correction: none

### Special details

**Table:**

Experimental. The low-temparture data was collected with the Oxford Cryosystem Cobra low-temperature attachment.
Geometry. All e.s.d.'s (except the e.s.d. in the dihedral angle between two l.s. planes) are estimated using the full covariance matrix. The cell e.s.d.'s are taken into account individually in the estimation of e.s.d.'s in distances, angles and torsion angles; correlations between e.s.d.'s in cell parameters are only used when they are defined by crystal symmetry. An approximate (isotropic) treatment of cell e.s.d.'s is used for estimating e.s.d.'s involving l.s. planes.
Refinement. Refinement of *F*^2^ against ALL reflections. The weighted *R*-factor *wR* and goodness of fit *S* are based on *F*^2^, conventional *R*-factors *R* are based on *F*, with *F* set to zero for negative *F*^2^. The threshold expression of *F*^2^ > σ(*F*^2^) is used only for calculating *R*-factors(gt) *etc*. and is not relevant to the choice of reflections for refinement. *R*-factors based on *F*^2^ are statistically about twice as large as those based on *F*, and *R*- factors based on ALL data will be even larger.

### Fractional atomic coordinates and isotropic or equivalent isotropic displacement parameters (Å^2^)

**Table:**

	*x*	*y*	*z*	*U*_iso_*/*U*_eq_	
O1	0.3014 (2)	0.54915 (8)	0.63643 (7)	0.0236 (3)	
O2	0.8876 (3)	0.51454 (8)	0.43322 (7)	0.0253 (3)	
O3	1.0904 (3)	0.35791 (8)	0.33767 (8)	0.0268 (3)	
O4	1.3439 (2)	0.46196 (8)	0.37431 (7)	0.0222 (3)	
C1	0.4708 (3)	0.48752 (10)	0.63241 (10)	0.0189 (3)	
C2	0.4861 (4)	0.43676 (10)	0.69963 (10)	0.0223 (3)	
H2A	0.3821	0.4459	0.7404	0.027*	
C3	0.6541 (3)	0.37447 (10)	0.70417 (10)	0.0213 (3)	
H3A	0.6623	0.3409	0.7481	0.026*	
C4	0.8161 (3)	0.35991 (10)	0.64334 (9)	0.0187 (3)	
C5	0.9937 (4)	0.29689 (10)	0.64975 (10)	0.0218 (3)	
H5A	1.0026	0.2646	0.6944	0.026*	
C6	1.1535 (3)	0.28219 (10)	0.59170 (10)	0.0226 (4)	
H6A	1.2696	0.2405	0.5970	0.027*	
C7	1.1400 (3)	0.33079 (10)	0.52406 (10)	0.0209 (3)	
H7A	1.2462	0.3199	0.4842	0.025*	
C8	0.9712 (3)	0.39479 (10)	0.51538 (9)	0.0182 (3)	
C9	0.8007 (3)	0.41091 (10)	0.57529 (9)	0.0171 (3)	
C10	0.6209 (3)	0.47439 (9)	0.57108 (9)	0.0177 (3)	
H10A	0.6048	0.5070	0.5268	0.021*	
C11	0.2839 (3)	0.60828 (10)	0.57358 (9)	0.0192 (3)	
H11A	0.2236	0.5798	0.5281	0.023*	
H11B	0.4396	0.6316	0.5618	0.023*	
C12	0.1165 (3)	0.67811 (10)	0.59753 (9)	0.0183 (3)	
C13	0.1697 (3)	0.76264 (10)	0.57956 (10)	0.0202 (3)	
H13A	0.3105	0.7757	0.5540	0.024*	
C14	0.0126 (3)	0.82699 (10)	0.59983 (10)	0.0212 (3)	
H14A	0.0485	0.8830	0.5875	0.025*	
C15	−0.1971 (3)	0.80841 (10)	0.63826 (10)	0.0219 (3)	
H15A	−0.3026	0.8517	0.6510	0.026*	
C16	−0.2495 (3)	0.72467 (11)	0.65769 (10)	0.0219 (3)	
H16A	−0.3884	0.7120	0.6845	0.026*	
C17	−0.0931 (3)	0.66001 (10)	0.63687 (10)	0.0198 (3)	
H17A	−0.1293	0.6040	0.6494	0.024*	
C18	0.9888 (3)	0.44716 (10)	0.44555 (10)	0.0187 (3)	
C19	1.1476 (3)	0.41483 (10)	0.38003 (9)	0.0189 (3)	
C20	1.4989 (4)	0.44233 (11)	0.30990 (10)	0.0232 (3)	
H20A	1.6051	0.4891	0.3010	0.035*	
H20B	1.4044	0.4326	0.2647	0.035*	
H20C	1.5899	0.3923	0.3214	0.035*	

### Atomic displacement parameters (Å^2^)

**Table:**

	*U*^11^	*U*^22^	*U*^33^	*U*^12^	*U*^13^	*U*^23^
O1	0.0271 (7)	0.0212 (5)	0.0224 (6)	0.0087 (5)	0.0058 (5)	0.0049 (5)
O2	0.0272 (7)	0.0212 (5)	0.0274 (6)	0.0057 (5)	0.0047 (6)	0.0037 (5)
O3	0.0274 (7)	0.0242 (6)	0.0287 (6)	−0.0045 (6)	0.0028 (6)	−0.0071 (5)
O4	0.0216 (6)	0.0225 (5)	0.0224 (6)	−0.0041 (5)	0.0035 (5)	−0.0030 (5)
C1	0.0202 (8)	0.0157 (6)	0.0207 (7)	0.0022 (6)	0.0002 (7)	0.0000 (6)
C2	0.0264 (9)	0.0206 (7)	0.0198 (7)	0.0030 (7)	0.0034 (7)	0.0013 (6)
C3	0.0265 (9)	0.0190 (7)	0.0185 (7)	0.0011 (7)	−0.0011 (7)	0.0026 (6)
C4	0.0216 (8)	0.0146 (6)	0.0198 (7)	−0.0004 (6)	−0.0027 (7)	−0.0008 (6)
C5	0.0270 (9)	0.0175 (7)	0.0210 (8)	0.0019 (7)	−0.0059 (7)	0.0012 (6)
C6	0.0238 (9)	0.0171 (7)	0.0268 (8)	0.0036 (6)	−0.0041 (7)	−0.0003 (6)
C7	0.0201 (8)	0.0192 (7)	0.0233 (8)	0.0015 (7)	−0.0001 (7)	−0.0027 (6)
C8	0.0189 (8)	0.0159 (6)	0.0199 (7)	−0.0004 (6)	−0.0004 (6)	−0.0014 (6)
C9	0.0188 (7)	0.0142 (6)	0.0183 (7)	−0.0013 (6)	−0.0013 (6)	−0.0020 (5)
C10	0.0196 (8)	0.0158 (7)	0.0177 (7)	0.0006 (6)	−0.0014 (6)	0.0006 (6)
C11	0.0218 (8)	0.0184 (7)	0.0175 (7)	0.0026 (6)	0.0002 (6)	0.0015 (6)
C12	0.0189 (8)	0.0187 (7)	0.0174 (7)	0.0017 (6)	−0.0029 (6)	−0.0014 (6)
C13	0.0218 (8)	0.0197 (7)	0.0192 (7)	−0.0006 (6)	−0.0003 (7)	0.0018 (6)
C14	0.0268 (9)	0.0156 (7)	0.0210 (7)	−0.0003 (7)	−0.0033 (7)	−0.0001 (6)
C15	0.0244 (9)	0.0202 (7)	0.0211 (8)	0.0046 (7)	−0.0018 (7)	−0.0032 (6)
C16	0.0193 (8)	0.0248 (8)	0.0216 (8)	0.0010 (7)	−0.0004 (7)	−0.0011 (6)
C17	0.0194 (8)	0.0177 (6)	0.0224 (8)	−0.0003 (6)	−0.0020 (7)	0.0009 (6)
C18	0.0183 (7)	0.0177 (7)	0.0201 (7)	−0.0021 (6)	0.0007 (6)	−0.0021 (6)
C19	0.0185 (8)	0.0167 (6)	0.0214 (7)	0.0007 (6)	0.0000 (6)	0.0012 (6)
C20	0.0202 (8)	0.0262 (8)	0.0232 (8)	−0.0001 (7)	0.0046 (7)	0.0009 (6)

### Geometric parameters (Å, °)

**Table:**

O1—C1	1.361 (2)	C8—C18	1.471 (2)
O1—C11	1.4387 (19)	C9—C10	1.422 (2)
O2—C18	1.222 (2)	C10—H10A	0.9300
O3—C19	1.204 (2)	C11—C12	1.505 (2)
O4—C19	1.332 (2)	C11—H11A	0.9700
O4—C20	1.452 (2)	C11—H11B	0.9700
C1—C10	1.375 (2)	C12—C17	1.391 (2)
C1—C2	1.418 (2)	C12—C13	1.399 (2)
C2—C3	1.363 (2)	C13—C14	1.389 (2)
C2—H2A	0.9300	C13—H13A	0.9300
C3—C4	1.414 (2)	C14—C15	1.385 (3)
C3—H3A	0.9300	C14—H14A	0.9300
C4—C5	1.411 (2)	C15—C16	1.392 (2)
C4—C9	1.432 (2)	C15—H15A	0.9300
C5—C6	1.370 (3)	C16—C17	1.392 (2)
C5—H5A	0.9300	C16—H16A	0.9300
C6—C7	1.405 (2)	C17—H17A	0.9300
C6—H6A	0.9300	C18—C19	1.534 (2)
C7—C8	1.391 (2)	C20—H20A	0.9600
C7—H7A	0.9300	C20—H20B	0.9600
C8—C9	1.437 (2)	C20—H20C	0.9600
			
C1—O1—C11	118.03 (13)	C12—C11—H11A	110.2
C19—O4—C20	115.80 (13)	O1—C11—H11B	110.2
O1—C1—C10	125.14 (14)	C12—C11—H11B	110.2
O1—C1—C2	113.69 (15)	H11A—C11—H11B	108.5
C10—C1—C2	121.15 (15)	C17—C12—C13	119.04 (16)
C3—C2—C1	119.68 (16)	C17—C12—C11	120.99 (15)
C3—C2—H2A	120.2	C13—C12—C11	119.97 (16)
C1—C2—H2A	120.2	C14—C13—C12	120.11 (17)
C2—C3—C4	121.24 (15)	C14—C13—H13A	119.9
C2—C3—H3A	119.4	C12—C13—H13A	119.9
C4—C3—H3A	119.4	C15—C14—C13	120.55 (15)
C5—C4—C3	120.65 (15)	C15—C14—H14A	119.7
C5—C4—C9	120.12 (16)	C13—C14—H14A	119.7
C3—C4—C9	119.22 (14)	C14—C15—C16	119.77 (16)
C6—C5—C4	121.56 (15)	C14—C15—H15A	120.1
C6—C5—H5A	119.2	C16—C15—H15A	120.1
C4—C5—H5A	119.2	C17—C16—C15	119.75 (17)
C5—C6—C7	119.30 (16)	C17—C16—H16A	120.1
C5—C6—H6A	120.4	C15—C16—H16A	120.1
C7—C6—H6A	120.4	C12—C17—C16	120.77 (15)
C8—C7—C6	121.45 (17)	C12—C17—H17A	119.6
C8—C7—H7A	119.3	C16—C17—H17A	119.6
C6—C7—H7A	119.3	O2—C18—C8	126.88 (16)
C7—C8—C9	120.19 (15)	O2—C18—C19	115.34 (15)
C7—C8—C18	116.73 (15)	C8—C18—C19	117.78 (14)
C9—C8—C18	122.94 (15)	O3—C19—O4	126.16 (16)
C10—C9—C4	118.63 (15)	O3—C19—C18	123.10 (16)
C10—C9—C8	124.02 (14)	O4—C19—C18	110.59 (14)
C4—C9—C8	117.35 (14)	O4—C20—H20A	109.5
C1—C10—C9	120.05 (14)	O4—C20—H20B	109.5
C1—C10—H10A	120.0	H20A—C20—H20B	109.5
C9—C10—H10A	120.0	O4—C20—H20C	109.5
O1—C11—C12	107.76 (13)	H20A—C20—H20C	109.5
O1—C11—H11A	110.2	H20B—C20—H20C	109.5
			
C11—O1—C1—C10	−3.3 (2)	C4—C9—C10—C1	1.9 (2)
C11—O1—C1—C2	175.28 (15)	C8—C9—C10—C1	−177.56 (15)
O1—C1—C2—C3	−177.83 (16)	C1—O1—C11—C12	−170.20 (14)
C10—C1—C2—C3	0.8 (3)	O1—C11—C12—C17	−42.1 (2)
C1—C2—C3—C4	0.7 (3)	O1—C11—C12—C13	138.51 (16)
C2—C3—C4—C5	178.08 (17)	C17—C12—C13—C14	−1.0 (3)
C2—C3—C4—C9	−0.9 (3)	C11—C12—C13—C14	178.34 (15)
C3—C4—C5—C6	−179.53 (16)	C12—C13—C14—C15	0.3 (3)
C9—C4—C5—C6	−0.6 (3)	C13—C14—C15—C16	0.9 (3)
C4—C5—C6—C7	−0.2 (3)	C14—C15—C16—C17	−1.4 (3)
C5—C6—C7—C8	1.4 (3)	C13—C12—C17—C16	0.5 (3)
C6—C7—C8—C9	−1.9 (3)	C11—C12—C17—C16	−178.85 (16)
C6—C7—C8—C18	174.04 (15)	C15—C16—C17—C12	0.7 (3)
C5—C4—C9—C10	−179.41 (15)	C7—C8—C18—O2	−166.30 (17)
C3—C4—C9—C10	−0.4 (2)	C9—C8—C18—O2	9.5 (3)
C5—C4—C9—C8	0.1 (2)	C7—C8—C18—C19	14.4 (2)
C3—C4—C9—C8	179.09 (15)	C9—C8—C18—C19	−169.81 (15)
C7—C8—C9—C10	−179.42 (15)	C20—O4—C19—O3	0.8 (2)
C18—C8—C9—C10	4.9 (3)	C20—O4—C19—C18	−174.75 (13)
C7—C8—C9—C4	1.1 (2)	O2—C18—C19—O3	−103.8 (2)
C18—C8—C9—C4	−174.57 (15)	C8—C18—C19—O3	75.6 (2)
O1—C1—C10—C9	176.33 (15)	O2—C18—C19—O4	71.91 (19)
C2—C1—C10—C9	−2.1 (3)	C8—C18—C19—O4	−108.69 (16)

### Hydrogen-bond geometry (Å, °)

**Table:**

*D*—H···*A*	*D*—H	H···*A*	*D*···*A*	*D*—H···*A*
C10—H10A···O2	0.93	2.28	2.896 (2)	124
C14—H14A···O2^i^	0.93	2.52	3.315 (2)	144
C20—H20B···O1^ii^	0.96	2.53	3.458 (2)	163
C7—H7A···Cg2^iii^	0.93	3.15	3.8529 (18)	134
C13—H13A···Cg3^iv^	0.93	3.13	3.8070 (19)	132
C17—H17A···Cg1^v^	0.93	3.12	4.0033 (17)	159

Symmetry codes:  (i) *x*−1/2, −*y*+3/2, −*z*+1; (ii) −*x*+3/2, −*y*+1, *z*−1/2; (iii) −*x*, *y*+1/2, −*z*+3/2; (iv) −*x*, *y*+3/2, −*z*+3/2; (v) *x*−1, *y*, *z*.
